# Toll-like receptor 3 regulates Zika virus infection and associated host inflammatory response in primary human astrocytes

**DOI:** 10.1371/journal.pone.0208543

**Published:** 2019-02-08

**Authors:** Chet Raj Ojha, Myosotys Rodriguez, Mohan Kumar Muthu Karuppan, Jessica Lapierre, Fatah Kashanchi, Nazira El-Hage

**Affiliations:** 1 Department of Immunology, Florida International University, Herbert Wertheim College of Medicine, Miami, Florida, United States of America; 2 National Center for Biodefense and Infectious Diseases, George Mason University, Manassas, Virginia, United States of America; University of Hong Kong, HONG KONG

## Abstract

The connection between Zika virus (ZIKV) and neurodevelopmental defects is widely recognized, although the mechanisms underlying the infectivity and pathology in primary human glial cells are poorly understood. Here we show that three isolated strains of ZIKV, an African strain MR766 (Uganda) and two closely related Asian strains R103451 (Honduras) and PRVABC59 (Puerto Rico) productively infect primary human astrocytes, although Asian strains showed a higher infectivity rate and increased cell death when compared to the African strain. Inhibition of AXL receptor significantly attenuated viral entry of MR766 and PRVABC59 and to a lesser extend R103451, suggesting an important role of TAM receptors in ZIKV cell entry, irrespective of lineage. Infection by PRVABC59 elicited the highest release of inflammatory molecules, with a 8-fold increase in the release of RANTES, 10-fold increase in secretion of IP-10 secretion and a 12-fold increase in IFN-β secretion when compared to un-infected human astrocytes. Minor changes in the release of several growth factors, endoplasmic reticulum (ER)-stress response factors and the transcription factor, NF-κB were detected with the Asian strains, while significant increases in FOXO6, MAPK10 and JNK were detected with the African strain. Activation of the autophagy pathway was evident with increased expression of the autophagy related proteins Beclin1, LC3B and p62/SQSTM1 with all three strains of ZIKV. Pharmacological inhibition of the autophagy pathway and genetic inhibition of the *Beclin1* showed minimal effects on ZIKV replication. The expression of toll-like receptor 3 (TLR3) was significantly increased with all three strains of ZIKV; pharmacological and genetic inhibition of TLR3 caused a decrease in viral titers and in viral-induced inflammatory response in infected astrocytes. We conclude that TLR3 plays a vital role in both ZIKV replication and viral-induced inflammatory responses, irrespective of the strains, while the autophagy protein Beclin1 influences host inflammatory responses.

## Introduction

Zika virus (ZIKV), an emerging infectious flavivirus has caused significant public health threat due to its potential association with neurodevelopmental disorders in fetus and neurodegenerative diseases in adult [1, 2]. The virus was first isolated from monkeys in the Zika forest of Uganda in 1947 and subsequently, the neutralizing antibody against the virus in human sera was reported in 1952 [3, 4]. The latest ZIKV outbreaks in French Polynesia (2013) and Brazil (2015–2016) linked ZIKV with increased prevalence of Guillain Barre Syndrome and microcephaly respectively [1, 2]. Increased cases of fetal malformations such as spontaneous abortion, stillbirth, hydrocephaly and microcephaly, and maternal placental insufficiency to miscarriage collectively known as congenital ZIKV syndrome (CZS) were reported as the consequences of ZIKV infection during gestation [5]. Genetic and phylogenetic studies reveal that ZIKV has evolved into 2 distinct lineages: the African lineage including the West African (Nigerian cluster) and the East African (MR766 prototype cluster) and the Asian lineage including Polynesian and Brazilian clusters. The virus most likely originated in East Africa and spread towards West Africa and Asia approximately 50–100 years ago [6, 7]. Genetic changes among the ZIKV lineage strains is attributed to the global spread of a new phenotype and the emergence of more neuro-virulent strains. Phylogenetic analysis and amino acid composition analysis of spatiotemporally different ZIKV strains isolated from human, mosquito and non-human primates have shown significant amino acid differences throughout the viral polyprotein [7]. Comparative studies of different ZIKV strains have shown inconsistent reports in terms of viral lethality, cellular infectivity, antiviral responses and genetic changes [7–11].

The primary route of virus transmission is by the bite of *Aedes* mosquitoes to humans, although, evidence for sexual transmission indicates human-to-human transmission is also possible [12, 13]. The molecular mechanisms of viral entry into brain cells and the associated pathology is still poorly understood. Current studies suggest that Tyro3 AXL MER (TAM) family of receptors are shown to be the primary entry factors for ZIKV in various cell types [14, 15]. However, controversial reports regarding their role in ZIKV infection within the brain necessitates further investigation using more than one spatiotemporal ZIKV strains [16, 17].

Besides neurons, glial cells, including astrocytes and microglia, are the most important targets for ZIKV pathology in the brain [18, 19]. Astrocytes, the most abundant glial cells are located in the vicinity of capillaries and are a key component of the blood-brain barrier (BBB). Therefore, astrocytes maybe the first targets encountered by ZIKV immediately after entering the central nervous system. Moreover, glial infection by ZIKV may cause BBB leakage leading to neuro-inflammation by releasing pro-inflammatory molecules [20]. ZIKV infection in the acute and convalescent phases induces secretion of pro-inflammatory molecules such as interleukin (IL)-1β, IL-6, interferon-γ-induced protein 10 (IP-10) or C-X-C motif chemokine ligand 10 (CXCL10), regulated on activation normal T cell expressed and secreted (RANTES) and macrophage inflammatory protein 1 alpha (MIP-1α); notably more chemokines than cytokines [21]. Activation of the toll-like receptor (TLR) pathway and subsequent upregulation of pro-inflammatory molecules secretion by glial cells is a key component of neuro-inflammation associated with many neurological disorders [22]. ZIKV is reported to activate TLR3 in human organoid cells and in murine neurospheres, leading to perturbation of genes related to neurodevelopment and reducing the organoid volume as seen in clinical microcephaly [23]. ZIKV strains MR766 and IbH30656, have also been reported to induce the autophagy pathway in fetal neuro-progenitor cells (fNSCs) and skin fibroblast [24, 25]. Autophagy is the process of lysosomal degradation of long lived proteins, cellular organelles and intracellular pathogens [26]. Both the autophagy and TLR3 pathways are important mediators of the innate immune response that are activated by ZIKV, with the TLR signaling pathway, being reported to induce the autophagy pathway [27–30].

The goal of the current study was to compare the level of infectivity and the release of inflammatory molecules among three isolated strains of ZIKV representing the African and Asian lineages and determine the mechanisms of their responses in primary human astrocytes. Our findings showed that significant differences in viral infection, cell viability, expression of stress response genes, secretion of inflammatory molecules and growth factors exists between the three strains. However, irrespective of viral strain, TAM receptors play a key role in ZIKV entry and the TLR3 pathway is an important player in ZIKV replication and in the release of inflammatory molecules in astrocytes, irrespective of viral strains.

## Materials and methods

### Zika virus propagation and isolation

Zika virus strains MR766 (Cat. VR84, ATCC, Manassas, VA, USA), R103451 (Cat. VR-1848, ATCC, Manassas, VA, USA) and PRVABC59 (Cat. VR-1843, ATCC, Manassas, VA, USA) were each propagated in Vero cells (Cat. CRL-1586, ATCC, Manassas, VA, USA) and/or mosquito cell line C6/36 (Cat. CRL-1660, ATCC, Manassas, VA, USA). Briefly, Vero cells or C6/36 cells were grown in T75 flasks with recommended growth media (EMEM supplemented with 10% FBS and 1% antibiotic/antimycotic solution) to > 80% confluency and infected with 4 ml of ZIKV (diluted in EMEM) at a multiplicity of infection (MOI) of 0.01. Culture flasks were incubated at 37°C and 5% CO_2_ for 2 hours with gentle rocking every 15 minutes, followed by addition of 4 ml of media and continuous incubation for 5 days. Supernatant was harvested after 5 days, centrifuged and filtered using 0.45-micron filter and aliquoted in 2.0 ml cryotubes [31]. Virus titer was determined by standard plaque assay and further amplified by propagating in C6/36 mosquito cells cultured in EMEM supplemented with 10% FBS at 28°C and 5% CO_2_.

### Viral quantification by plaque assay

Vero cells were infected with ten-fold dilution of ZIKV stock or the supernatants from infected/treated cells for 1 hour to allow adsorption and then washed with phosphate buffer saline (PBS). Cells were overlaid with culture media (EMEM supplemented with 2% FBS) containing equal volume of 3.2% carboxymethylcellulose (CMC) and incubated for 5 days at 37°C. Cells were fixed and stained with 1% crystal violet solution prepared in 20% formaldehyde, 30% ethanol and 50% PBS for 1 hour. Stained cells were washed with water to remove excess crystal violet, left to dry overnight, and the lysis plaques were quantified by stereomicroscope. The viral titer was expressed as plaque forming units (PFU) per ml of the stock.

### Cell cultures, treatment and ZIKV infection

Human primary astrocytes (Cat. 1800, ScienCell, Carlsbad, CA, USA) were grown and maintained in astrocyte medium supplemented with 2% FBS, 1% astrocyte growth supplement (AGS) and 1% antibiotic/antimycotic solution (ScienCell, Carlsbad, CA, USA). Small interfering RNA (siRNA) against Beclin1 (Cat. sc-29797) or TLR3 (Cat. sc-36685) were purchased from Santa Cruz Biotechnology (Santa Cruz, CA, USA). Rapamycin was purchased from Sigma-Aldrich (St. Louis, MO, USA) and used at a concentration of 2.5μM. 3-methtladenine (3-MA) and chloroquine (CQ) were used at concentration of 1μM and 40μM, respectively. The concentrations were based on the dose response viability assay performed in our previous studies [32, 33]. The TLR3/dsRNA complex inhibitor (a thiophenecarboxamidopropionate compound) and TLR3 agonist (Poly I:C) were purchased from Millipore Sigma and Sigma Aldrich, respectively. To infect with each viral strain, viral stock was diluted in the appropriate media based on cell types. Infection dose or the MOI was determined from the number of cells to be infected and titer of viral stocks. As higher viral dose had toxic effects to the primary cells, an MOI of 0.1 was used for all studies unless mentioned otherwise.

### Immunocytochemistry

Viral infectivity with each viral strain was measured by fluorescent immunolabeling. Briefly, cells were fixed in 4% paraformaldehyde, permeabilized with 0.1% Triton X-100, and blocked in 10% milk/0.1% goat serum. Human astrocytes were immunolabeled with mouse anti-GFAP antibody (Cat. MAB360, Millipore, Boston, MA, USA) and rabbit anti-ZIKV-E antibody (Cat. GTX133314, Genetex, Irvine, CA, USA). Immunoreactivity was visualized with secondary antibodies from molecular probes (Carlsbad, CA, USA). 4′,6-diamidino-2-phenylindole (DAPI) was used to label cell nuclei. The Images were analyzed using an inverted fluorescence microscope with a 560 Axiovision camera (Zeiss, Germany).

### Assessment of cell viability

Cell viability was determined by trypan blue dye exclusion method. Briefly, cells were harvested using enzyme free cell dissociation buffer after 24, 48, 72 and 96 hours’ post-infection (hpi) with each strain of ZIKV. After gentle centrifugation, the resulting cell pellets were re-suspended in respective culture media. Equal volume of 0.40% trypan blue dye (Cat. 145–0013, Bio-Rad, Hercules, CA, USA) was added to each cell suspension. Total number of viable and nonviable cells were counted by using TC20 automated cell counter (Bio-Rad, Hercules, CA, USA). Cell viability was expressed as percentages of viable cells out of total cells.

### Inflammation and growth factor antibody array

Expression profiles of inflammatory molecules and growth factors were screened by human inflammation antibody array C3 and human growth factor antibody array C1 (Ray Biotech, GA, USA), respectively using cell culture supernatant from astrocytes infected with different strains of ZIKV. Briefly, antibody array membranes were incubated with sample for overnight at 4°C, followed by biotinylation and streptavidin labelling. Chemiluminescence signals were detected by ChemiDoc imaging system (Bio-Rad, Hercules, CA, USA).

### ELISA

Secretion of interleukin (IL)-6 and -8, RANTES, monocyte chemoattractant protein 1 (MCP-1), IP-10 and interferon beta (IFN-β) by glial cells were measured in culture supernatants of human astrocytes infected with three different strains of ZIKV (MR766, R103451 and PRVABC59) for different time points (24, 48,72 and 96 hours) by ELISA (RANTES Cat. DY278-05, MCP-1 Cat. DY279-05, IL-8 Cat. DY208-05, IL-6 Cat. DY206-05 and IFN-β Cat. 41410–1, R&D Systems, Minneapolis, MN, USA). The optical density (O.D.) was read at A450 on a Synergy HTX plate reader (BioTek, Winooski, VT, USA).

### Immunoblotting

Whole cell lysates were prepared in RIPA buffer (Thermo Scientific, Waltham, MA, USA) supplemented with a mixture of protease and phosphatase inhibitors and separated by SDS-PAGE for immunoblotting. Primary antibodies against Beclin1 (Cat. ab62557, 1:1500), Abcam, Cambridge, MA, USA), LC3B (Cat. 12741, 1:1000), Cell Signaling, Danvers, MA, USA), P62/SQSTM1 (Cat. ab91526 (1:2000), Abcam, Cambridge, MA, USA), ZIKV anti-E (Cat. GTX133314, 1:1000) Genetex, Irvine, CA, USA), TLR3 (Cat. sc-28999, 1:200), TLR4 (Cat sc-30002, 1:200), TLR5 (Cat. sc-10742, 1:200), MyD88 (Cat. sc-74532, 1:200), PERK (Cat. sc-377400, 1:200), IRE1α (Cat. sc-390960, 1:200), ATF6α (Cat. sc166659, 1:200), DDIT3 (Cat. sc-7351, 1:200) and β actin (Cat. sc-47778, 1:200) (Santa Cruz Biotechnology, Santa Cruz, CA, USA) were followed by incubation with a respective secondary antibody conjugated to horseradish peroxidase (Millipore, Billerica, MA, USA) used at specified dilution. The immunoblots were exposed to SuperSignal West Femto Substrate (Thermo Scientific, Waltham, MA, USA) and visualized using a ChemiDoc imaging system (Bio-Rad, Hercules, CA, USA).

### mRNA expression profiling

Human astrocytes were infected with three strains of ZIKV (MR766, R103451 and PRVABC59) for 24 hours. RNA was isolated using the RNeasy Mini Kit (Qiagen, Valencia, CA, USA). Purity of the RNA (O.D. 260/280 nm absorbance ratio of at least 2.0) was measured by a microspot RNA reader (Synergy HT Multi-Mode Microplate Reader from BioTek) and 0.5 μg of RNA was used for cDNA synthesis using Qiagen’s RT First Strand Kit (Cat. 330401, Qiagen, Valencia, CA, USA) as per the manufacturer’s protocol after eliminating genomic DNA. Then, the synthesized cDNA was mixed with RT SYBR Green/ROX PCR master mix (Cat. 330520, Qiagen, Valencia, CA, USA) to run RT-PCR in 96 well plate according to manufacturer’s protocol. Ct values were determined after setting the threshold and baseline manually and were uploaded into the data analysis online software provided on the manufacturer’s website (http://pcrdataanalysis.sabiosciences.com/pcr/arrayanalysis.php) according to the manufacturer’s instructions to determine the relative expression of each gene compared with the expression in control.

### Real time RT-PCR

ZIKV RNA was extracted from cell culture supernatant using QIAamp Viral RNA mini kit (Qiagen, Valencia, CA, USA). Cellular RNA was extracted by RNeasy Mini Kit (Qiagen, Valencia, CA, USA). iTaq universal SYBR Green one-step PCR kit (Bio-Rad, Hercules, CA, USA) was used for determining Zika viral RNA titer using 10μM of primers (Forward: 5’-CCGCTGCCCAACACAAG-3’ and Reverse: 5’-CCACTAACGTTCTTTTGCAGACAT-3’) purchased from Sigma (Sigma-Aldrich, St. Louis, MO, USA). PCR conditions consisted of reverse transcription for 10 min at 50°C, a hold step at 95°C for 1 min followed by 40 amplification cycles of 95°C for 10 secs and 60°C for 30 sec. The specificity of the amplified products was verified by melting curve analysis and agarose gel electrophoresis. The Standard curve was prepared from 10-fold dilutions of previously quantified ZIKV stock solution with known titer. Viral titer in the solution was expressed as RNA copies equivalent of log_10_ PFU/ml.

### RNA interference: Silencing of Beclin1 and TLR3 in human astrocytes

Human primary astrocytes were transfected with siRNA against TLR3 or with siRNA against Beclin1 for 24–48 hours using Lipofectamine 2000 (Invitrogen, Carlsbad, CA, USA) or Fugene HD (Promega, Madison, WI, USA) for Beclin1 and TLR3 knockdown. siRNA and transfection reagent (ratio, 1:2) were pre-incubated for 20 min in OptiMEM medium before addition to the seeded cells. Knockdown of targets were confirmed in cell lysates by western blot for the protein expression of TLR3 and Beclin1. CRISPR-cas9 method of silencing was also utilized for enhanced silencing of Beclin1 in human astrocytes. Guide RNA (gRNA) for *BECN1* and Cas9 enzyme were mixed in the ratio of 1.3:1 and incubated at room temperature for 5 minutes to make RNP- complex, followed by incubation with transfusion reagent (Fugene HD, Promega, Madison, WI, USA) for 20 minutes. The resulting RNP-transfection solution was added to the seeded cells and incubated for at least 24 hours before ZIKV infection.

### Statistical analysis

Results are reported as mean±SEM of 3–6 independent experiments each with more than three samples. Data were analyzed using analysis of variance (ANOVA) followed by appropriate post hoc test for multiple comparisons (GraphPad Software, Inc., La Jolla, CA, USA). An alpha level (p value) of < 0.05 was considered significant.

## Results

### Levels of viral infection differ between the Asian and African ZIKV strains in human astrocytes

**Fig 1:**
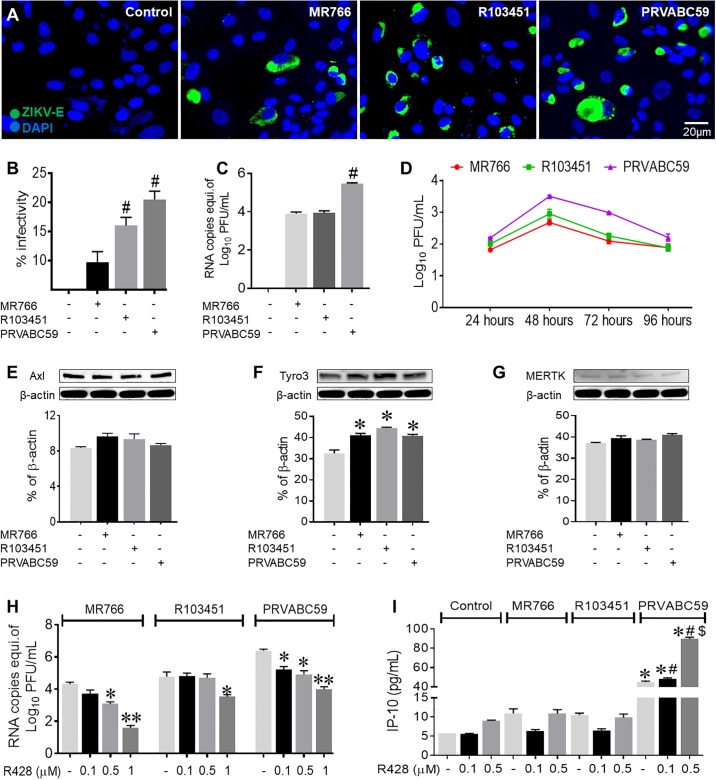
Asian and African strains of ZIKV have differential levels of infectivity in human astrocytes. (A) Representative images of human astrocytes infected (MOI of 0.1) with three different strains of ZIKV (MR766, R103451 and PRVABC59) for 24 hours and immunolabeled with ZIKV envelope antibody. DAPI was used for nuclei staining. Images were captured using an inverted fluorescence microscope with a 560 Axiovision camera (Zeiss, Germany). (B) Data analysis of fluorescence images after manual counting of the number of cells infected with virus shows differential infectivity levels among the three strains of ZIKV. (C) Intracellular ZIKV RNA copies as measured by RT-PCR using ZIKV specific primers at 24 hours post infection (hpi). (D) Viral titers in supernatant of ZIKV-infected astrocytes were measured by plaque assay at the indicated time points. (E- G) Western blot analysis of the cell lysate of astrocytes uninfected and infected with three strains of ZIKV, for putative receptors AXL, Tyro3 and MERTK. (H) ZIKV infectivity in the human astrocytes treated with increasing concentration of AXL inhibitor (R428) as measured by RT-PCR. (I) Interferon gamma-induced protein 10 (IP-10) measured by ELISA using supernatant from human astrocytes treated with R428 followed by ZIKV infection. Mock (PBS) infected human astrocytes were used as control and the infection dose of ZIKV was at an MOI of 0.1. Data are presented as the mean ± SEM from 3–5 independent experiments. (*p< 0.05 Vs Control, # p < 0.05 Vs MR766).

ZIKV permissiveness in primary human astrocytes was determined by immunofluorescence RT-PCR and western blot (Fig 1A–1C and S1A Fig). Human astrocytes were infected with either the Asian (R103451 and PRVABC59) or the African (MR766) strains of ZIKV. Twenty-four hours post infection (hpi), infectivity was detected by measuring the expression level of ZIKV envelope protein by immunofluorescence. Envelope protein was detected in astrocytes infected with each strain of ZIKV although the expression levels, representing infectivity, were significantly different among the strains (Fig 1A and 1B). Similar results were observed with human microglia (S1B and S1C Fig). Infectivity was confirmed by measuring ZIKV RNA by RT-PCR from the cellular RNA extract and envelope protein by western blot using the cell lysate of the infected astrocytes, which validated the immunofluorescence data (Fig 1C and S1A Fig). Overall, the data showed higher level of infection with the Asian strains (PRVABC59 and R103451) when compared to the African strain of ZIKV in astrocytes. We further validated the kinetics of viral replication in human astrocytes, using plaque assay in the supernatants collected at 24, 48, 72 and 96 hpi from ZIKV-infected astrocytes. The highest viral titers for all three strains of ZIKV was detected at 48 hpi, after which the titers were decreased with time (Fig 1D). We detected higher levels of intracellular viral RNA copies with RT-PCR when compared to number of viral progeny secreted in the supernatant detected with plaque assay, indicating that the numbers of active viral progeny release in the supernatant might not correlate with the amount of intracellular viral RNA. That was further evidenced by the observation that PRVABC59 strain showed significantly higher RNA titers compared to the other two strains at 24 hpi, whereas the differences in virions secretion as measured by plaque assay was witnessed only at 48 hours (Fig 1C and 1D). We observed similar results in microglia except for those infected with MR766 strain, where the highest titer was reported at 72 hpi (S1C Fig). In spite of high infectivity of astrocytes (about 10–25% with MOI of 0.1), we detected relatively low number of viral progeny (in the range of 2–4 log_10_ PFU/mL) in comparison to microglia (S1D Fig), suggesting that human astrocytes are efficiently infected but with limited capacity to produce viral progeny.

To determine a potential mode of viral entry in human glial cells, we investigated the expression of putative host receptors utilized by ZIKV, as reported by others [14, 15]. Members of the TAM receptors, Tyro3, AXL and MERTK were detected in human astrocytes, but surprisingly infection with any of the three ZIKV strains had no effect on protein expression levels of AXL, although slight increase in the expression of Tyro3 was observed with both Asian and African strains (Fig 1E–1G). To examine the role of TAM receptors in ZIKV entry into human astrocytes the pharmacological inhibitor, R428, was used to induce a dose-dependent decrease in the expression levels of the AXL receptor. Downregulation of the host receptor correlated with a decrease in ZIKV (MR766 and PRVABC59) cell entry, while viral entry decreased in the Honduras strain (R103451) of ZIKV was detected only at the highest concentrations of inhibitor (Fig 1H, S1E and S1F Fig). Similar results were shown by siRNA mediated silencing of AXL in primary human astrocytes (S1H and S1I Fig). Overall the data suggest strain dependent differences in the extent of inhibition of ZIKV entry by TAM inhibitors specifically AXL. Concurrent decrease in IFN-β signaling was also detected in the supernatant of viral infected astrocytes treated with R428, suggesting that viral entry leads to induction of host immune responses (S1G Fig). However, IP-10, a biomarker for severity of viral infection was enhanced by both pharmacological and genetic inhibition of AXL in astrocytes infected with PRVABC59 (Fig 1I and S1I Fig). Infection or viral entry with all three strains were detected even after the treatment with high dose of R428, suggesting involvement of an alternative or secondary mechanism of ZIKV entry into astrocytes. Nevertheless, the data indicate that AXL receptor play an important role in ZIKV infection and host immune response. Since we observed significant upregulation of Tyro3 receptor expression by ZIKV, we next inhibited Tyro3 by c-Met specific inhibitor (BMS777607) in the astrocytes. However, inhibition of Tyro3 had no effect on viral infection or replication as well as on inflammatory molecule (IP-10) secretion, indicating that ZIKV cellular entry is independent of Tyro3 (S1J and S1K Fig).

### Asian strains of ZIKV induce higher levels of inflammatory molecules in human astrocytes

**Fig 2:**
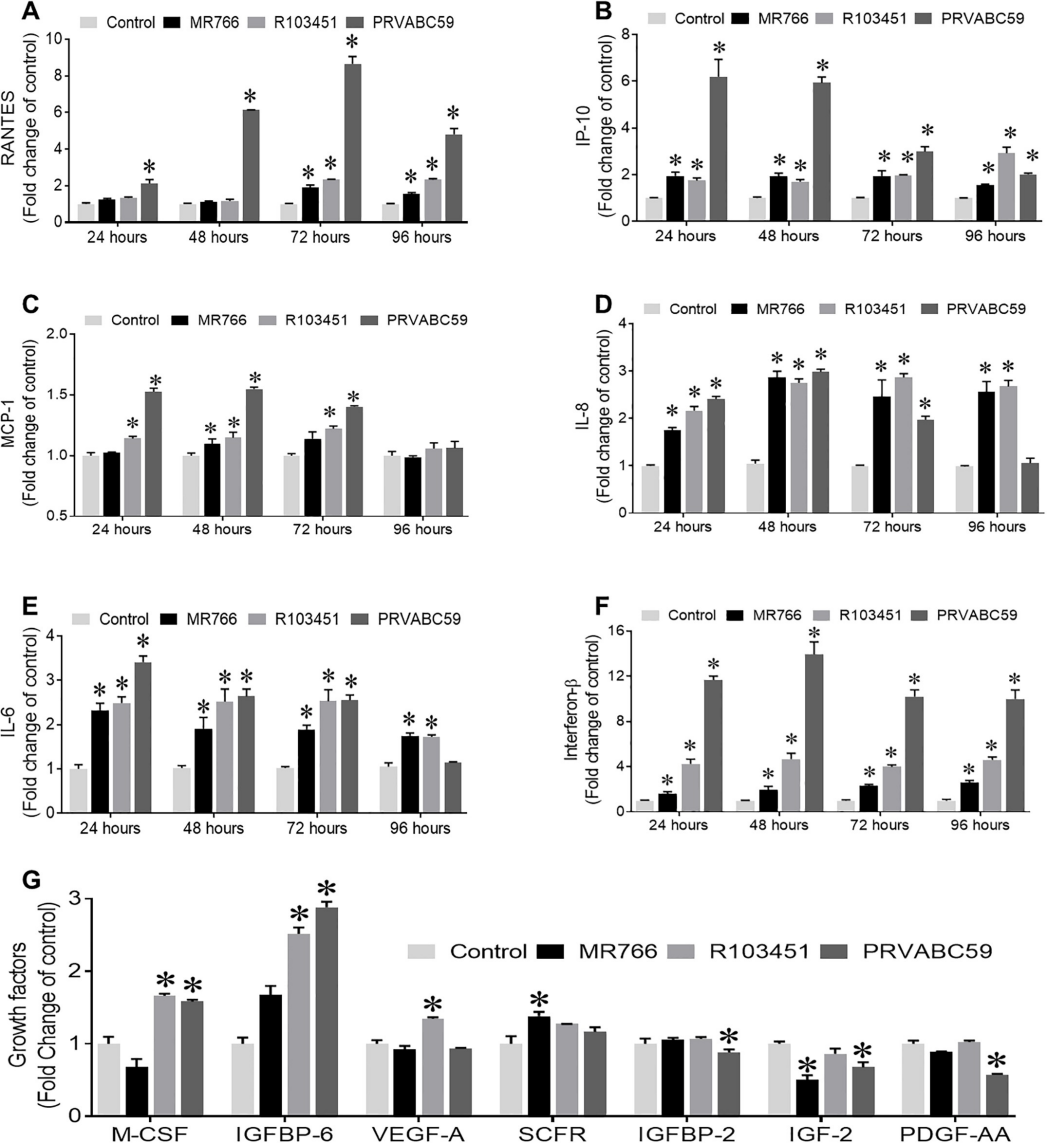
Differential induction of inflammatory molecules and type I interferon secretion by three spatiotemporally different ZIKV in human astrocytes. (A-F) Human astrocytes were infected with three different strains of ZIKV (MR766, R103451 and PRVABC59) at an MOI of 0.1. Supernatants were collected at the indicated time points and secretion of indicated inflammatory molecules (A-E) and IFNα (data not shown) and IFNβ (F) were measured by ELISA and expressed as fold change of control. (G) Expression of growth factors after 48 hpi were measured by growth factor antibody array. Mock (PBS)infected human astrocytes were used as control. Data are presented as the mean ± SEM from at least three independent experiments. (*p<0.05 Vs control).

As glial cells are the principal cell types involved in the release of neuro-inflammatory molecules, a key marker of viral pathology in the brain, we examined changes in the secretion of inflammatory molecules by astrocytes infected with different strains of ZIKV (MOI of 0.1). Human inflammatory antibody array was performed using the supernatant collected from the ZIKV infected astrocytes at 48 hpi. Different inflammatory molecules including IL-6, IL-1α, IL-4, IP-10, RANTES and transforming growth factor β1 (TGF-β1) were induced in human astrocytes by all three strains of ZIKV. The Puerto Rican strain (PRVABC59) had a more robust effect on the release of inflammatory factors (S2A Fig). We confirmed our results using individual ELISA for MCP-1, RANTES, IL-8, IP-10, and IL-6. Both the Asian and African strains of ZIKV induced the secretion of MCP-1, RANTES, IP-10, IL-8 and IL-6 (Fig 2A–2E). Interestingly, the Puerto Rican strain (PRVABC59) had the largest effect causing about 8-fold increase in RANTES, a 6-fold increase in IP-10, a 1.5-fold increase in MCP-1 and a 3.5-fold increases in IL-8 and IL-6 when compared to un-infected control. With the exception of IL-8, peak levels of the inflammatory molecules were detected at 48 or 72 hpi, which correlated with the detected viral titers (Fig 1D). The kinetics of cytokines/chemokines secretion showed that the PRVABC59 strain caused a rapid cytokine and chemokine response which subsided with time. However, the MR766 the strain induced a slower, persistent cytokine response. In human microglia, the inflammatory antibody array showed increased secretion of RANTES, IP-10, MIP-1α and MIP-1B with the PRVABC59 strains which was supported by ELISA (S2B–S2E Fig). Since, type 1 IFN is considered an indicator of early antiviral response by host cells to restrict virus spread [34], we measured type 1 IFN levels (IFN-α and IFN-β) using ELISA and detected secretion of IFN-β in human astrocytes infected with all three ZIKV strains (Fig 2F), whereas IFN-α was undetectable (data not shown). Although, all three strains showed a significant increase in the secretion of IFN-β levels, the Puerto-Rican strain (PRVABC59) induced more than a 12-fold increase in secretion with a peak reaching at 48 hpi (Fig 2F), suggesting a more rigorous anti-viral response. The sustained IFN-β response by human astrocytes against the PRVABC59 strain of ZIKV over the period of 96 hours’ correlates with the suppression of viral titers.

Since, ZIKV has been linked to growth retardation and microcephaly which might be caused by alteration in the expression or release of growth factors, we analyzed changes in human growth factors using an antibody array. Cell culture supernatant from human astrocytes infected with the three different strains of ZIKV was analyzed. With the exception of insulin-like growth factor binding protein-6 (IGFBP-6) which showed a 2-fold increase in expression, minor increases in the release of the growth factors monocyte-colony stimulating factor (M-CSF), epidermal growth factor (EGF), basic fibroblast growth factor (bFGF) and decreases in insulin-like growth factor-2 (IGF-2), insulin-like growth factor binding protein 2 (IGFBP-2) and platelet derived growth factor-AA (PDGF-AA) (Fig 2G) were detected in astrocytes infected with the Puerto Rican strain (PRVABC59). Although we observed slight changes in the expression levels of several growth factors with both the Asian and African strains, these were not significant (Fig 2G).

### Pathological and molecular alterations by African and Asian strains of ZIKV in human astrocytes

**Fig 3:**
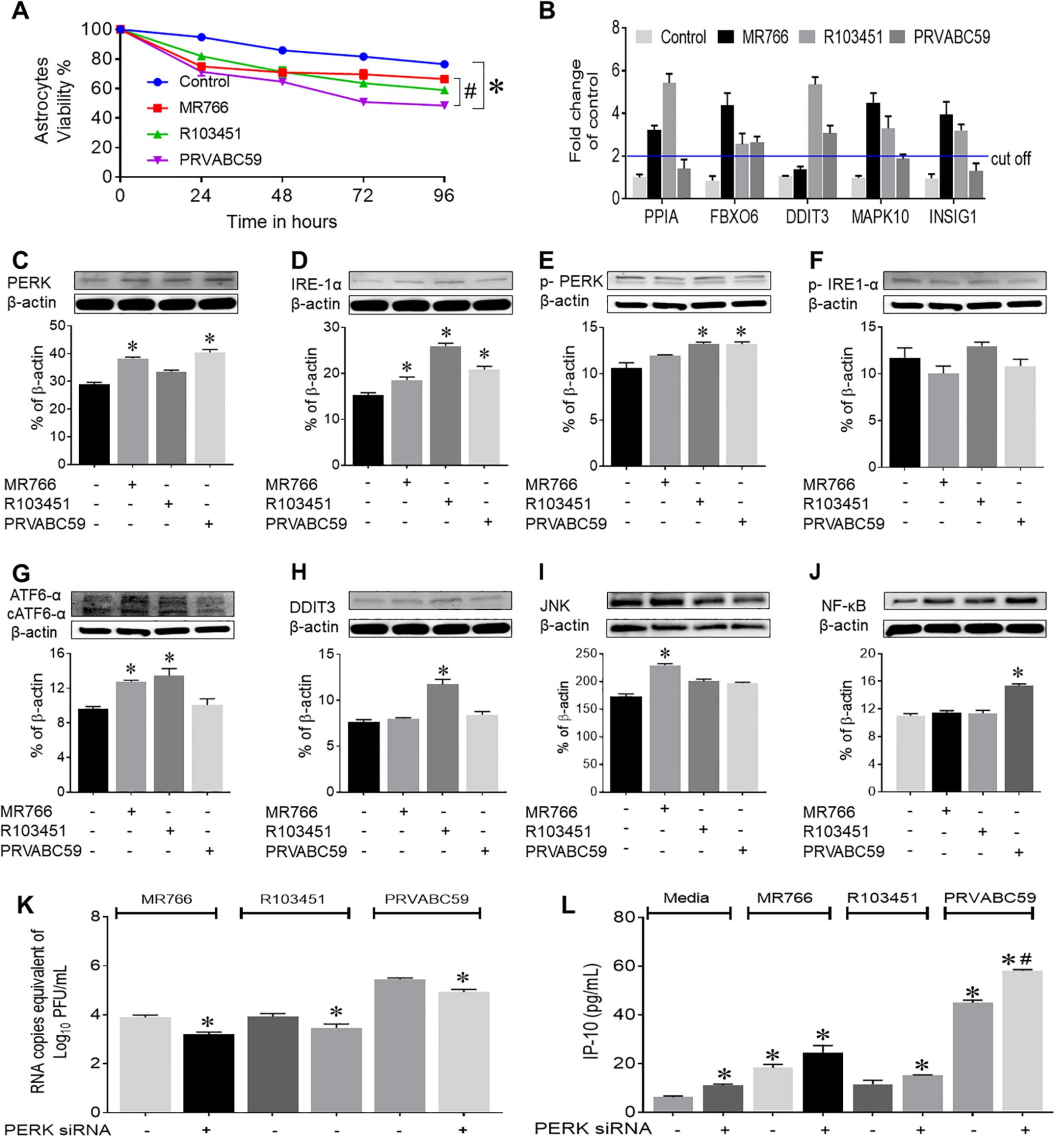
ZIKV induces ER stress, DNA damage and cell death in human astrocytes. (A) Astrocytes viability was determined by trypan blue dye exclusion method at different time point after infection with ZIKV. (B) Human astrocytes were infected with ZIKV (MR766, R103451 and PRVABC59) at an MOI of 0.1. After 48 hpi, RNA was extracted from cell lysates and RT-PCR arrays were performed to determine RNA expression of genes involved in human unfolded protein response pathway. Genes significantly altered by ZIKV infection are presented in the graph. (C-H) Western blot analysis from cell lysates (collected at 48 hpi in RIPA buffer) from uninfected and ZIKV infected human astrocytes showed expression of proteins involved in the unfolded protein response, including, PERK, IRE-1α, phosphorylated PERK, phosphorylated IRE-1α, full length and cleaved ATF6α, and DDIT3. (I and J) JNK and NF-κB protein expression by the African and the Asian strain respectively as measured by western blot with cell lysates of human astrocytes collected after 48 hpi. (K-L) Human astrocytes were transfected with siRNA against *PERK* and infected with ZIKV for 48 hours. Viral titer was measured by RT-PCR (K) and IP-10 levels were measured by ELISA (L). Mock (PBS) infected human astrocytes were used as control and the infection dose of ZIKV was at an MOI of 0.1. Data are presented as the mean ± SEM from three independent experiments. (*p<0.05 Vs control, ^#^p<0.05 Vs ZIKV alone).

In order to determine additional pathological consequences associated with ZIKV infection, we measured cell death after 24, 48, 72 and 96 hours post infection in human astrocytes. Cell viability was significantly decreased after 48 hpi with all three strains of ZIKV, while the Puerto Rican strain was significantly more lethal to astrocytes in comparison to the African strain MR766 (Fig 3A). A Similar decrease in viability was noted in microglia infected with Asian and African strains of ZIKV, however, in neurons, the Honduras strain (R103451) induced the highest cell death than other two strains (S3A and S3B Fig). Overall, the rate of infectivity among each viral strain of ZIKV correlated with cell death in human astrocytes, however, the Puerto-Rican strain (PRVABC59) was more infectious and replicated more vigorously which correlated with high levels of inflammation and cell death.

Exploring the mechanism of ZIKV-mediated pathology, we investigated endoplasmic reticulum (ER) stress and related pathways. Since flaviviruses including ZIKV, replicate in close association with the ER, we reasoned that ZIKV replication mediates ER stress responses that could potentially be linked to high levels of inflammation and cell death [35]. We measured changes in mRNA expression of genes involved in the unfolded protein response pathway using gene expression profile arrays from RNA isolated after infection with the different strains of ZIKV in human astrocytes. Infection with the Asian strains (R103451 and PRVABC59) showed increased expression of the DNA damage inducible transcript 3 (*DDIT3)/CHOP* and peptidyl-prolyl *cis-trans* isomerase A (*PPIA*) genes, while infection with the African strain caused increased expression of fork-head box 06 (*FOX06*), mitogen activated protein kinase -10 (*MAPK10*), Insulin inducible gene 1 (*INSIG1*) and *PPIA* genes (Fig 3B). In terms of cellular function, it has been reported by others that PPIA binds to unfolded proteins including viral proteins, the *FOX06* gene is involved in DNA damage and cell cycle regulation, the *MAPK10* (also called Janus kinase-3 or *JNK3*) gene, regulates multiple biological processes including stress induced neuronal apoptosis while *INSIG1* regulates lipid metabolism [36–39].

We further analyzed the activation and phosphorylation of the proteins involved in the ER-stress and UPR pathways by western blot. The Uganda strain (MR766) caused upregulation of the protein kinase R (PKR)-like endoplasmic reticulum kinase (PERK), inositol requiring enzyme 1α (IRE-1α) and activating transcription factor-6 α (ATF6α) proteins, while infection with the Honduran strain induced expression of IRE-1α and ATF6α. The Puerto-Rican strain induced PERK and IRE-1α. Increased phosphorylated PERK (p-PERK) was detected with the Asian strain of ZIKV, however, phospho-IRE-1α was not increased with any of the viral strains. The cleaved form of ATF6α was slightly increased with Uganda (MR766) and Honduras (R103451) strains (Fig 3C–3G). Interestingly, infection with the Honduras strain (R103451) also induced protein expression of DDIT3/CHOP (Fig 3H), which is a member of the CCAAT/enhancer-binding protein (C/EBP) family of transcription factor which is activated by ER stress to promote apoptosis.

We observed upregulation of the *MAPK10* gene in cells infected with the African strain (MR766), and given that MAPK signaling pathway is known to induce inflammation, we examined the expression level of JNK, a major component of MAPK signaling, by western blot. Infection with MR766 caused moderate induction in the expression levels of the JNK protein (Fig 3K), which also acts as downstream effector molecule and modulator for the UPR pathway [40]. In addition, the transcription factor nuclear factor‐κB (NF‐κB), a regulator of the immediate early host pathogen inflammatory and cell survival responses [41, 42] was examined. We detected a significant upregulation in protein expression of NF-κB in human astrocytes infected with the Puerto-Rican strain (PRVABC59) (Fig 3L). Activation of NF-κB is followed by degradation of associated inhibitory molecule IκBα and nucleation localization of NF-κB for transcriptional regulation of various genes. Therefore, we examined whether NF-κB is localized to nucleus by immunofluorescence staining of infected astrocytes with NF-κB (p65) along with GFAP and DAPI. We observed increased nuclear localization of NF-κB in the astrocytes infected with each of the ZIKV strains (S3C Fig). Overall, infection with ZIKV induces several key proteins involved in the UPR, MAPK and the NF-κB pathways as well as genes involved in DNA damage responses that may be involved in the regulation of ZIKV-induced inflammatory molecules and cell death, respectively. We observed only a minor induction in the genes and proteins of the abovementioned pathways, therefore we did not pursue these pathways further. Moreover, the ER stress and related pathways are the consequences of viral infection and replication inside the cells and may not necessarily control the viral replication and associated pathology. In order to investigate the role of UPR pathway in ZIKV infection and associated immune inflammatory response, we downregulated UPR using siRNA against PERK, which has been reported to influence ZIKV mediated pathology in neurons [43]. The siRNA mediated downregulation of PERK resulted in a significant decrease in ZIKV replication in human astrocytes (Fig 3K) with corresponding increase in IP-10 secretion (Fig 3L).

### Increased expression of autophagy proteins in ZIKV-infected human astrocytes

**Fig 4:**
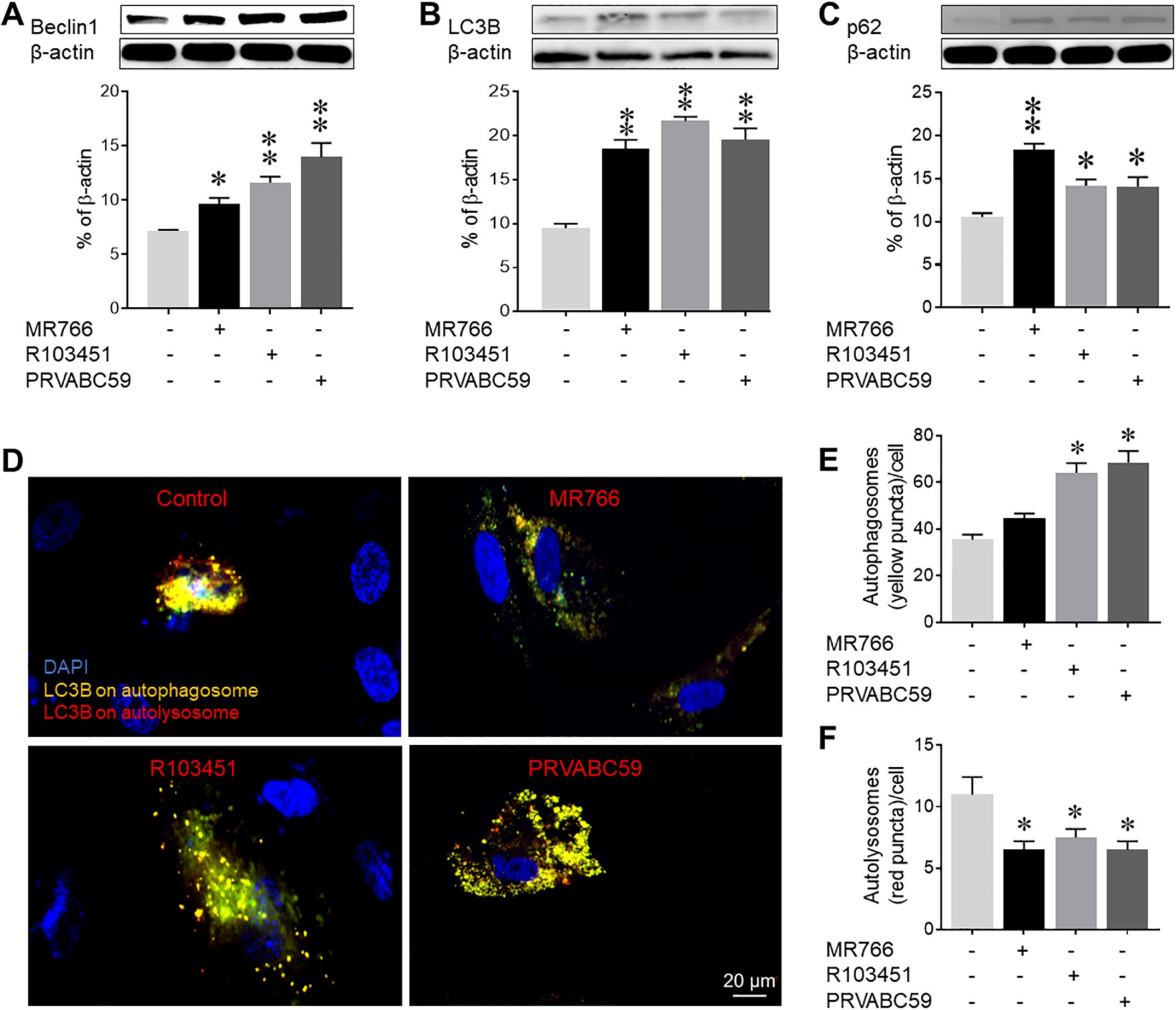
ZIKV induces autophagosome formation and blocks autophagosome-lysosome fusion. (A-C) Human astrocytes were infected with three strains of ZIKV (MOI = 0.1), cell lysates collected at 48 hpi and protein expression of Beclin1, LC3B and p62/SQSTM1 were measured by western blot. (D) Representative images of uninfected and ZIKV (three strains) infected human astrocytes shows yellow and red LC3B puncta as determined by a cell-based assay for LC3B. (E and F) The numbers of yellow and red puncta from the cell-based assay were counted per cells. Presence of both green and red (yellow) fluorescence indicates autophagosome, while red fluorescence only indicates autolysosome. Mock (PBS) infected human astrocytes were used as the control. Data are presented as the mean ± SEM from at least 3 independent experiments (*p<0.05 Vs control, and **p<0.01 Vs control).

Along with UPR activation, flavivirus mediated ER stress may also lead to activation of the autophagy pathway, which has been an attractive target for various viruses [44, 45]. In fact, infection with ZIKV induces autophagosome formation in different cell types including the human fibroblast, cytotrophoblast and fetal neuroprogenitor stem cells (fNSC) [25, 46]. As such, the autophagy pathway may be used by ZIKV as a mechanism associated with the pathology in glial cells. To this end, we measured expression levels of key proteins involved in the autophagy pathway by western blot (Fig 4A–4C). Infection of astrocytes with MR766, R103451 and PRVABBC59 showed about 1.3, 1.6 and 1.9-fold, increase respectively, in Beclin1 expression (Fig 4A). LC3-II, a marker for autophagosome formation [32, 47] was increased by ~2-fold (Fig 4B) while p62/SQSTM1, a ubiquitin binding scaffold protein that facilitate binding of ubiquitinated protein to autophagosome and a marker for autophagosome degradation, was upregulated by ~1.5 fold (Fig 4C) in astrocytes infected with all three strains of ZIKV. Analysis of the autophagy pathway using a cell-based assay that utilizes the tandem mRFP-GFP tagged LC3 reporter plasmid for fluorescence analysis (Fig 4D–4F) showed similar results with upregulation in autophagosome formation in astrocytes infected with each of the ZIKV strain. The tandem mRFP-GFP LC3 assay employs the differential pH stability of green fluorescent protein (GFP) and red florescent protein (RFP) in acidic environment of lysosome. We measured a 2-fold increase in autophagosome formation in astrocytes infected with R103451 and PRVABC59 strains of ZIKV, as indicated by the yellow puncta (Fig 4E) and a decrease in the maturation or lysosomal pathway in cells infected with each of the strains (Fig 4F), as detected by a decrease in red puncta. Overall, we showed activation of the autophagy pathway in cells infected with each strain of ZIKV. Based on our western blot and cell-based assay findings, it is possible that infection by ZIKV induces the initial activation and maturation stages of the autophagy pathway and yet blocks the autophagosome-lysosome fusion and degradation. This, in turn, prompted us to further examine the role of this pathway in ZIKV infectivity and pathology in primary human astrocytes.

### Modulation of the autophagy pathway showed no significant changes in viral replication

**Fig 5:**
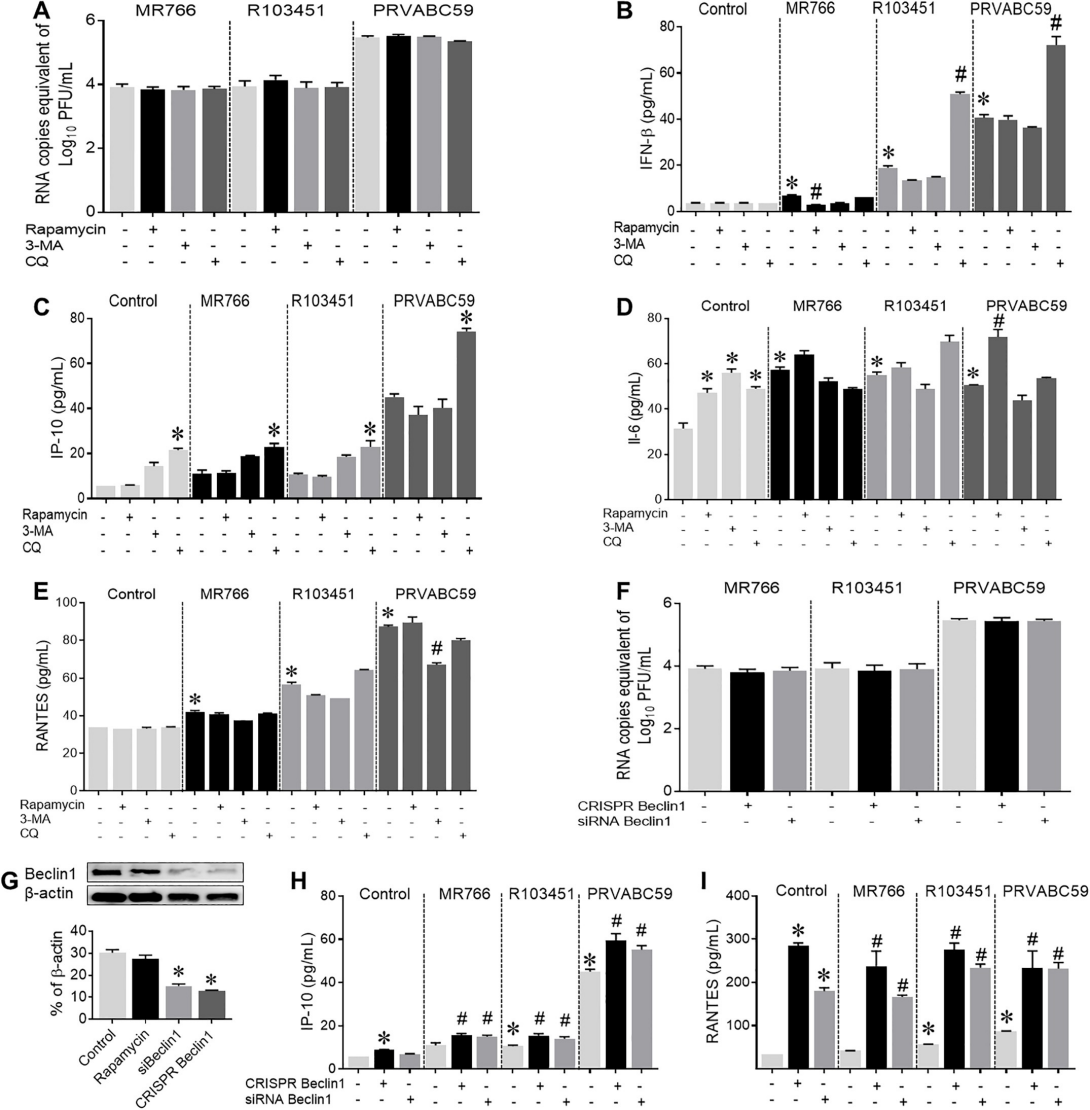
Modulation of Autophagy pathway shows no significant changes in viral replication. The autophagy pathway was modulated before ZIKV infection either by using pharmacological inducer (rapamycin) or inhibitors (3-MA or chloroquine) or genetic silencing of *Beclin1* (siRNA or CRISPR-Cas9). (A) Viral titers were measured by RT-PCR using cell supernatants collected after infection with three different strains of ZIKV and indicated treatments. (B-E) Cell supernatant after 48 hpi were used to measure IFN-β, IP-10, IL-6 and RANTES by ELISA. (F) ZIKV RNA copies measured by RT-PCR after silencing *Beclin1* with siRNA or CRISPR-Cas9 system. (G) Detection of Beclin1 protein by western blot analysis confirmed silencing efficiency of the siRNA. (H and I) Secretion of IP-10 and RANTES measured by ELISA after 48 hpi with indicated treatments. Mock (PBS) infected human astrocytes were used as the control. Data are presented as the mean ± SEM from at least 3 independent experiments. (*p<0.05 Vs control, ^#^ p<0.05 Vs ZIKV alone).

The role of autophagy in mediating ZIKV replication and associated pathogenesis in glial cells has not yet been explored. Here we use pharmacological and genetic approaches to modulate the autophagy pathway in ZIKV-infected human astrocytes. Cell supernatant was collected and the viral titer (replication) of each strain was determined by RT-PCR and confirmed by plaque-forming assay. Induction of the autophagy pathway with rapamycin (an mTOR inhibitor) caused a slight but insignificant increase in the viral titer of R103451 (Fig 5A) and a decrease in MR766-induced secretion of IFN-β (Fig 5B). No further changes were detected in the viral titer of MR766 and PRVABBC59 nor in the secretion of IP-10, RANTES and IL-6 (irrespective of strain) (Fig 5C–5E). Blocking the formation of autophagosome with the inhibitor, 3-methyladenine (3-MA) had no effect on ZIKV replication and the associated inflammatory response (Fig 5A–5E). On the contrary, exposure with chloroquine (CQ), a lysomotropic agent that inhibits lysosomal degradation, caused a slight decrease in the replication of PRVABC59 and caused an increase in R103451-induced secretion of IFN-β (Fig 5A and 5B). Similarly, exposure to CQ enhanced the ZIKV-induced secretion levels of IP-10 in astrocytes infected with each of the viral strains (Fig 5E).

Next, we examined the role of Beclin1 in the pathology of ZIKV since we have previously reported that Beclin1 can regulate replication and viral induced neuro-inflammation in glial cells infected with the Human Immunodeficiency virus [48–50]. We silenced the expression of Beclin1 by CRISPR/cas9 and siRNA against Beclin1 which was confirmed by western blot (Fig 5F). Although, all three strains of ZIKV induced the expression of Beclin1 (Fig 4A), silencing with CRISPR-Cas9 had no further effect on the viral replication in astrocytes infected with ZIKV (Fig 5F). Interestingly, silencing Beclin1 (both siRNA and CRISPR mediated) further enhanced ZIKV-induced secretion of RANTES, IL-6 and IP-10 (Fig 5G–5I and S4 Fig). Overall, the fluctuations in the release of inflammatory molecules did not correlate with the amount of viral titer present in astrocytes suggest that ZIKV-induced inflammatory responses are not mediated by autophagy pathway.

### Toll-like receptor-3 regulates ZIKV replication and host inflammatory responses

**Fig 6:**
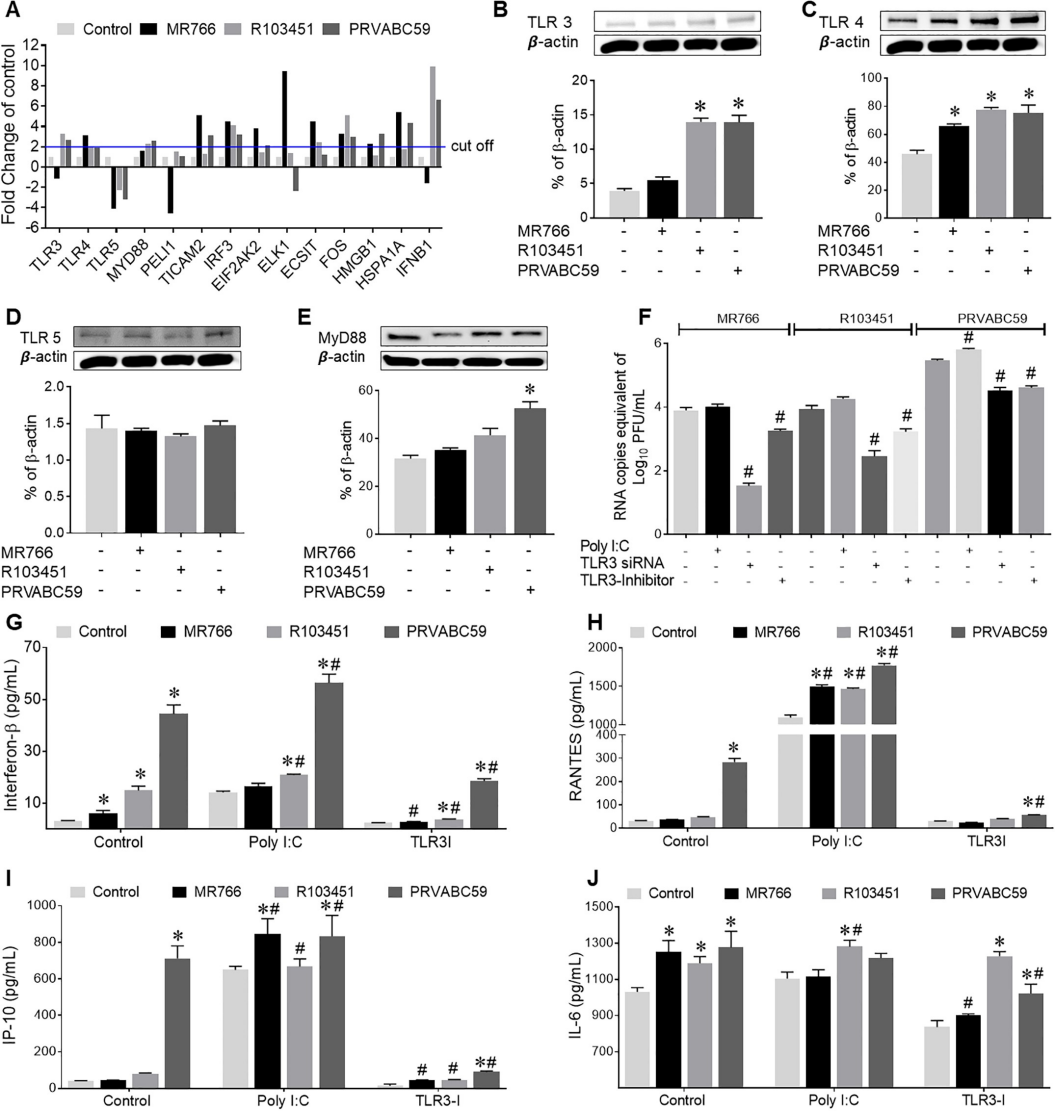
TLR3 regulates ZIKV replication and secretion of inflammation molecules. (A) Human astrocytes were infected with ZIKV (MR766, R103451 and PRVABC59) at an MOI of 0.1. After 48 hpi, RNA was extracted from cell lysates and RT-PCR array assays were performed to determine RNA expression of genes involved in the human TLR pathway. A 2-fold change in gene expression was used as the cut-off value. (B-D) Detection of TLR3, TLR4 and TLR5 expression levels by western blot after 48 hpi. (E) Protein expression levels of the TLR adaptor protein MyD88 after infection with ZIKV-PRVABC59. (F-J) Human astrocytes were infected with three different strains of ZIKV (MR766, R103451 and PRVABC59) at an MOI of 0.1. Sets of experiment included astrocytes treated with 5μg/mL of Poly I: C (TLR3 agonist), or with TLR3 silenced with siRNA or inhibited with a thiophenecarboxamidopropionate compound (TLR3/dsRNA complex inhibitor). After 48 hpi, RNA was extracted from the supernatant and RT-PCR using ZIKV specific primers was performed to determine viral titers (F). Cell supernatants were used to perform ELISA to detect levels of IL-6, RANTES and IFN-β (G-J). Mock (PBS) infected human astrocytes were used as the control. Data are presented as the mean ± SEM from 3 independent experiments. (*p<0.05, Vs control, ^#^ p<0.05 Vs ZIKV alone).

In addition to the autophagy pathway, toll-like receptors (TLRs) are essential signaling pathways involved in the innate immune defense mechanism, as they recognize and respond to pathogens. Despite this, little is known about TLR-mediated ZIKV replication and pathology in glial cells. Modulation of the TLR pathway by ZIKV-infection in primary human astrocytes was examined by gene expression profiling (Fig 6A) and protein expression was confirmed by western blot (Fig 6B–6D). mRNA expression profile showed upregulation in the levels of TLR3 with Asian strains, upregulation in TLR4 with all three strains and down-regulation of TLR5 with all three strains of ZIKV. In addition, the ETS transcription factor-1 (ELK1), which is activated by MAPK signaling and involved in multiple functions ranging from neuronal differentiation to chromatin remodeling [51], was increased with MR766, however was decreased by PRVABC59 (Fig 6A). Similarly, mRNA expressions of genes involved in TLR signaling pathway such as TICAM2, IRF3, ECSIT, EIF2K2 and HSPA1A were induced by the Uganda strain, whereas IRF3, ECSIT, FOS and IFN-β were upregulated by the Puerto-Rican strain. On the other hand, Pellino-1 (PEl1), a regulator of TLR signaling pathway [52], was downregulated with the Uganda strain (MR766) (Fig 6A). Together with mRNA expression, TLR3 protein expression levels were also upregulated by around 3-fold in astrocytes infected with either R103451 or PRVABC59 (Fig 6B), TLR4 protein expression was upregulated by approximately 2-folds with all three strains (Fig 6C), while minor changes were detected in TLR5 expression level (Fig 6D). An adaptor protein involved in TLR signaling pathway, MyD88, was slightly increased in astrocytes infected with the Puerto Rican strains (PRVABC59) (Fig 6A and 6E).

Since TLR3 was the highest up-regulated protein, we investigated whether this receptor is involved in ZIKV replication and associated inflammatory responses in astrocytes. Genetic silencing with siRNA against the *TLR3* gene or with pharmacological inhibitor (a thiophenecarboxamidopropionate compound) showed a decrease in viral replication in cells infected with each of the three strains (Fig 6F) with the African (MR766) strain showing the greatest decrease in viral titer after silencing with siRNA. Likewise, inhibition of TLR3 caused a significant decrease in ZIKV-induced IFN-β along with RANTES, IP-10 and IL-6 secretion by the Puerto Rican strain (PRVABC59) in astrocytes (Fig 6G–6J). On the other hand, exposure with the TLR3 agonist, Poly I: C, slightly increased the viral titers and significantly increased the secretion of IFN-β, RANTES and IP-10 in the supernatant of ZIKV-infected astrocytes (Fig 6F–6I). Similarly, a decrease in viral titers and secretion of inflammatory molecules was observed in human microglia by downregulation of TLR3 (S5A–S5D Fig), suggesting that TLR3 regulates ZIKV replication as well as ZIKV-induced secretion of inflammatory molecules in glial cells.

Since the TLR signaling pathway has been shown to induce the autophagy pathway [27] via adaptor proteins MyD88 or TICAM, we next investigated a potential link between TLR3 and the autophagy pathway in the context of ZIKV infection and pathology. Attenuation of TLR3 by genetic silencing resulted in a decrease in the expression levels of MyD88, TICAM1, IRF3 and Beclin1 (S6A–S6D Fig); suggesting a potential interaction between the proteins involved in TLR signaling and autophagy pathway or inhibition of a common protein necessary for the induction of both the pathways. Interestingly, silencing TLR3 caused a further increase in the protein expression levels of p62/SQSTM1 (S6E Fig) suggesting that accumulation of autophagy markers is likely to result from a blockage in the completion of basal autophagy rather than up-regulation of the pathway (based low expression of Beclin1) [53]. Overall, our data suggests that TLR3 mediates basal autophagy in ZIKV-infected astrocytes, although TLR3 mediated ZIKV replication may be independent of the autophagy pathway. Although TLR3 signaling is MyD88 independent, a downregulation of MyD88 along with TICAM1 with TLR3 silencing in human astrocytes occurred.

## Discussion

The association of recent ZIKV outbreaks with congenital birth defects and neurological disorders has brought great attention to this global health problem. There is a dire need for the development of a safe and effective vaccine against this neurotropic virus. The mechanisms behind the neuropathologies mediated by ZIKV are still unclear. More severe neurological consequences of ZIKV have been reported with the Asian lineage of ZIKV indicating differential ability between the Asian and the African lineage of ZIKV to infect brain cells and develop neurological complications. Our study aimed to describe the glial-pathogenesis of ZIKV among three different strains of ZIKV and explore the underlying mechanisms of their response. To our knowledge, this is the first report that compares the glial-pathogenesis among three spatiotemporally different ZIKV strains. Based on the isolation history, genomic sequence analysis, phylogenetic analysis and geographical distribution of the virus, we selected three viral strains representing both Asian and African lineage of virus.

We reported that the Asian lineage of ZIKV, specifically the Puerto-Rican strain (PRVABC59) showed higher infection which correlated with higher levels of pro-inflammatory molecules secretion and increased cell death in glial cells. A recent study has reported that Asian (ZIKV-Br) and African strains (MR766) of ZIKV are almost equally infective to human brain cortical astrocytes inducing secretion of IP-10, IL-6, IL-8, and RANTES [54]. Contrasting to our findings, Huang et al. observed higher infectivity with African strain (MR766) than the Asian strain (PRVABBC59) in human fetal astrocytes [55]. The discrepancy among these studies might be associated with the viral strains, passage numbers, sources of the astrocytes, MOI of virus used and other factors. Nevertheless, all of the studies conclude that ZIKV strains differentially infect astrocytes and induce antiviral responses with typically higher levels of chemokines. Our study indicated that ZIKV enters host cells via TAM receptors however alternative mechanism of cellular entry was not ruled out. We reported that inhibition of AXL caused a decrease in ZIKV infection and IFN-β secretion, although AXL is reported to antagonize type 1 IFN signaling to promote ZIKV infection instead of facilitating viral entry in astrocytes [56].

Glial cells have a primary role in the innate immunity in the brain by recognition of pathogens and their clearance [57]. Here, we focused on astrocytes due to their abundance in the brain, their direct linkage with neurons and their role in synaptic support, axonal guidance and control of the BBB [58]. Herein, we reported significant increase in the inflammatory molecules, RANTES, MCP-1, IP-10, IL-8 and IL-6 with ZIKV infection in glial cells. IP-10, an important biomarker of severity in many infectious disease and anti-flaviviral response in the CNS was previously reported to be induced by ZIKV and other flaviviruses [59–62]. Notably, IP-10 is strongly implicated in Guillain Barre Syndrome, indicating that ZIKV mediated neuronal injury might be mediated by elevated levels of IP-10. In this study, the Asian strains of ZIKV, specifically the Puerto-Rican strain (PRVABBC59), which is more likely to cause neurodegenerative disorders, showed a very high level (up to 6-fold) of IP-10 secretion by astrocytes. The pro-inflammatory molecules; IL-6, IL-8, RANTES and MCP-1, which recruit and activate other cell types to the infection site and amplify the inflammatory cascades, were augmented by all three stains of ZIKV in astrocytes. ZIKV induced IFN-β in general, however, the secretion levels were strain dependent with the Puerto-Rican strain inducing around a 14-fold increase. Although inflammatory molecules and IFNs are secreted as a host defense mechanism to attenuate viral burden, the high levels of inflammatory molecules secreted by brain cells may disrupt the BBB and facilitate further spread of virus in CNS [60, 63]. Cytokine kinetics in our study mimics the kinetics reported in clinical cases of ZIKV where RANTES, IL-6, IP-10 and IFN-β were elevated both in acute and convalescent phases of ZIKV infection, albeit higher levels were detected during the acute phase. On the other hand, a higher level of IL-8 was detected during the convalescent phase than the acute phase of ZIKV infection. Moreover, elevations in chemokines were more pronounced than the cytokines [64]. In line with our study, different kinetics of anti-viral responses in astrocytes infected with Asian (H/PF/2013) and African strain (HD78788) of ZIKV has been reported by Hamel et.al. Rapid antiviral response as shown by increased expression of several inflammatory molecules including IP-10, RANTES, IL-6, and IFN-β within 6 hpi was induced with Asian strains when compared to the African strain which induced delayed response [65]. Although the changes were not significant, we observed minor changes in the growth factors, M-SCF, IGFBP-6, IGFBP-2, IGF-2 and PDGF-AA in ZIKV infected astrocytes, which may contribute to ZIKV associated neurodevelopmental disorders.

ZIKV utilizes several cell signaling pathways including the UPR pathway through the three arms of the signaling pathway, which may facilitate viral persistence or replication inside the cells [66]. Extensive rearrangement of ER membrane and collapse of ER cisternae juxtaposed with viral particles and virus-induced vesicles has been recorded by others using electron tomography, indicating the role of ER and related pathways in ZIKV infection [54]. However, in our study, silencing of one of the arm of UPR showed minimal effect on ZIKV replication. We further looked into the MAPK and NF-κB pathways as additional possible mechanisms given that they have been reported to be involved in the apoptosis and cytokine response mediated by similar flavivirus, Dengue virus (DENV) [67]. We observed that depending on the viral strains, ZIKV either induces the MAPK pathway or the NF-κB pathway, which might also explain the differential levels of secretion of inflammatory molecules by the three spatiotemporally different ZIKV strains. NF-κB is also reported to repress the expression of DDIT3, possibly to limit the ER stress induced cell death [68, 69].

The role of autophagy in ZIKV replication is intensively being studied. Accumulation of autophagosome in various cells including human fetal neuronal stem cells, fibroblast, placental endothelial cells and trophoblasts infected with ZIKV have recently been reported [70, 71]. Autophagy, as an innate defense mechanism, has the potential to restrict ZIKV replication, however, viruses including flaviviruses evolve specific mechanisms to overcome autophagic degradation [72]. Our data further confirmed the induction of the autophagy pathway with possible blockage of autophagic clearance by ZIKV, irrespective of viral strains. However, modulation of the autophagy pathway had minimal effect on ZIKV replication and ZIKV-induced secretion of inflammatory molecules. Silencing of Beclin1 caused further enhancement in the secretion of RANTES, IP-10 and IL-6, which may be related to any cellular mechanism involving Beclin1 other than the autophagy pathway.

TLR3 which recognizes viral dsRNA, has an important role in flaviviral replication and associated pathology [63]. TLR3 knock out mice were less vulnerable to severe West Nile Virus (WNV) infection, cytokine production, neuronal injury and viral replication in brain [63]. On the other hand, a protective role of TLR3 in WNV and DENV infection has also been reported by other groups [28, 73]. Until recently, very few if any studies have evaluated the role of TLR3 pathway in ZIKV associated neuropathology [74]. Recently, a study reported that ZIKV-mediated over-activation of TLR3 leads to the depletion of neural progenitor cells in human cerebral organoids [23, 74]. In our current study, we found that TLR3 was induced by the Asian strains of ZIKV in astrocytes. Similar to our findings, previous studies have shown the upregulation of TLR3 by ZIKV in various cell types including human astrocytes, skin fibroblast and mouse neurospheres [23, 65, 75]. We reported that silencing or pharmacological inhibition of TLR3 caused significant decrease in RNA copies of both Asian and African strains ZIKV with correlated decreases in viral-induced inflammatory response, supporting the role of TLR3 in ZIKV replication and associated inflammatory immune response, irrespective of the strains. Studies have shown that TLR3 modulates virus permissiveness, replication, disease severity, immunogenicity in various other viruses including DENV, WNV, Influenza A virus, HIV-1, Herpes simplex, suggesting that TLR3 function is not specific to ZIKV [73, 76–79].

Both the autophagy and the TLR3 pathways are important mediators of the innate immune response and we report that both the pathways are induced by ZIKV. Activation of TLR3 was previously reported to induce the autophagy pathway [27–30]. MyD88 and TRIF (TICAM1), two adaptor proteins involved in the TLR signaling pathway can interact with the autophagy protein, Beclin1 with this interaction inhibiting the binding between Beclin1 and the anti-apoptotic protein, B-cell lymphoma 2 (BCL-2). Dissociation of Beclin1 from BCL-2 by activated MyD88 or TRIF facilitates induction of the autophagy pathway, while autophagic processing of viral RNA in turn may activate TLR3 signaling pathway [30]. TLR3 pathway is independent of MyD88, however MyD88 was downregulated upon silencing of TLR3. A possible explanation might be siTLR3 suppressed the viral titers to the levels which may not be sufficient to induce MyD88. A decrease in expression of MyD88 and TRIF with concomitant reduction in the protein expression levels of Beclin1 by TLR3 silencing in the current study indicates a potential link between TLR pathway and the autophagy pathway. Increase in p62/SQSTM1 with TLR3 silencing in ZIKV infected astrocytes might also suggest the role of p62/SQSTM1 to facilitate clearance of viral proteins via the ubiquitin proteasomal system [53].

In summary, we report differences in infectivity rate and in the secretion of inflammatory molecules in human glial cells infected with three different spatiotemporal strains of ZIKV. These differences may account for differential pathology in the viral strains and clinical manifestations in affected populations. Our data showed that the AXL receptor is important host molecules for ZIKV entry. Different levels of inhibition in viral entry between the three strains of ZIKV with AXL inhibitor suggest the possibility of another mechanism of ZIKV entry depending on the viral strains. ZIKV strains differentially activated the autophagy and the TLR pathway, two important mechanisms involved in regulating the host innate immune defense. Although modulation of the autophagy pathway induced changes in ZIKV replication, the changes in viral titer did not correlate with the changes in the inflammatory molecules. On the contrary, attenuating TLR3 caused a decrease in viral replication that correlated with the decreased amount of in viral replication, making TLR3 a potential target for ZIKV therapeutic approach.

## Supporting information

S1 Fig ZIKV infects human microglial cells and utilizes AXL receptor for cell entry. (A) Western blot shows expression of ZIKV-envelope protein in primary human astrocytes infected with three strains of ZIKV (MR766, R103451 and PRVABC59) (B) Representative images of human microglia infected with ZIKV (MR766, R103451 and PRVABC59) for 24 hours and immunolabeled with ZIKV envelope antibody. (C) Percentage infectivity of ZIKV in human microglia measured by immunofluorescence. (D) Viral titers in supernatant of ZIKV infected microglia measured by plaque assay at 24, 48, 72 and 96 hpi. (E and F) TAM inhibitor reduced ZIKV infection in human astrocytes. (E) Representative images of human astrocytes infected with ZIKV (PRVABC59) in presence or absence of R428. (F) Infectivity of human astrocytes measured by immunofluorescence staining. (G) IFN-β response measured by ELISA from cell supernatant of infected astrocytes exposed to increasing concentrations of R428. (H) Viral titers measured by RT-PCR in supernatant of ZIKV infected astrocytes with or without siRNA against AXL. (I) IP-10 secretion measured by ELISA using supernatant of ZIKV infected astrocytes with or without siRNA against AXL. (J) Viral titers measured by RT-PCR using supernatant of ZIKV infected astrocytes with or without exposure of Tyro3 inhibitor (BMS777607). (K) IP-10 secretion measured by ELISA using supernatant of ZIKV infected astrocytes with or without exposure of Tyro3 inhibitor (BMS777607). Mock (PBS) infected cells were used as control and the infection dose of ZIKV was at an MOI of 0.1. Data are presented as mean ± SEM from at least three independent experiments. (* p< 0.05 Vs Control, **p< 0.01 Vs Control ^#^ p < 0.05 Vs ZIKV alone).
(TIF)

S2 Fig Inflammatory molecules secreted by human astrocyte and microglia infected with three different strains of ZIKV. (A) Inflammation was measured using human Cytokine Antibody Array from culture supernatant of ZIKV infected glia. Expression levels are presented as fold increase from control. (B-E) Inflammatory molecules secreted by human microglia infected with three different strains of ZIKV measured by antibody array (B) and ELISA (C-E). Mock (PBS) infected cells were used as control and the infection dose of ZIKV was at an MOI of 0.1. Data are presented as mean ± SEM from at least three independent experiments. (*p< 0.05 Vs Control).
(TIF)

S3 Fig Cell viability and NF-κB nuclear localization. (A) Viability of human microglia at 24, 48, 72 and 96 hpi measured by trypan blue exclusion method. (B) Viability of neurons determined by time lapse image analysis. (C) Immunofluorescence staining of primary human astrocytes with NF-κB, GFAP and DAPI shows both nuclear and cytoplasmic localization of NF-κB. Error bars shown as mean ± SEM from 3–5 separate experiments. Mock (PBS) infected cells were used as control and the infection dose of ZIKV was at an MOI of 0.1. Data are presented as mean ± SEM from at least three independent experiments. (*p< 0.05 Vs Control).
(TIF)

S4 Fig IL-6 levels from astrocytes with RNA interference for Beclin1. Secretion of IL-6 measured by ELISA using human astrocytes supernatant after 48 hours post infection. Data are presented as mean ± SEM from at least three independent experiments. Mock (PBS) infected cells were used as control and the infection dose of ZIKV was at an MOI of 0.1. Data are presented as mean ± SEM from at least three independent experiments. (*p< 0.05 Vs Control).
(TIF)

S5 Fig TLR3 regulates ZIKV replication and inflammatory response in human microglia. (A) ZIKV titers measured by RT-PCR after 48hpi and TLR3 silencing. (B-D) Inflammatory molecules measured by ELISA after 48 hpi with or without siRNA against TLR3. Mock (PBS) infected cells were used as control and the infection dose of ZIKV was at an MOI of 0.1. Data are presented as mean ± SEM from at least three independent experiments. (*p< 0.05 Vs Control).
(TIF)

S6 Fig TLR3 silencing downregulates Beclin1 and upregulates p62/SQSTM1. (A-C) Expression of MyD88, TICAM1 and IRF3 (A), Beclin1 (B) and p62/SQSTM1 (C) with and without siRNA against *TLR3* as measured by western blot. Mock (PBS) infected cells were used as control and the infection dose of ZIKV was at an MOI of 0.1. Data are presented as mean ± SEM from at least three independent experiments. (*p<0.05 Vs control, ^#^ Vs ZIKV alone).
(TIF)

S1 File Supplemental materials and methods. (DOCX)
