# Supplementary figures and images for: Toll-like receptor 3 regulates Zika virus infection and associated host inflammatory response in primary human astrocytes
Source: PLoS One. 2019 Feb 8;14(2):e0208543. doi: 10.1371/journal.pone.0208543 (PMC6368285; doi:10.1371/journal.pone.0208543)

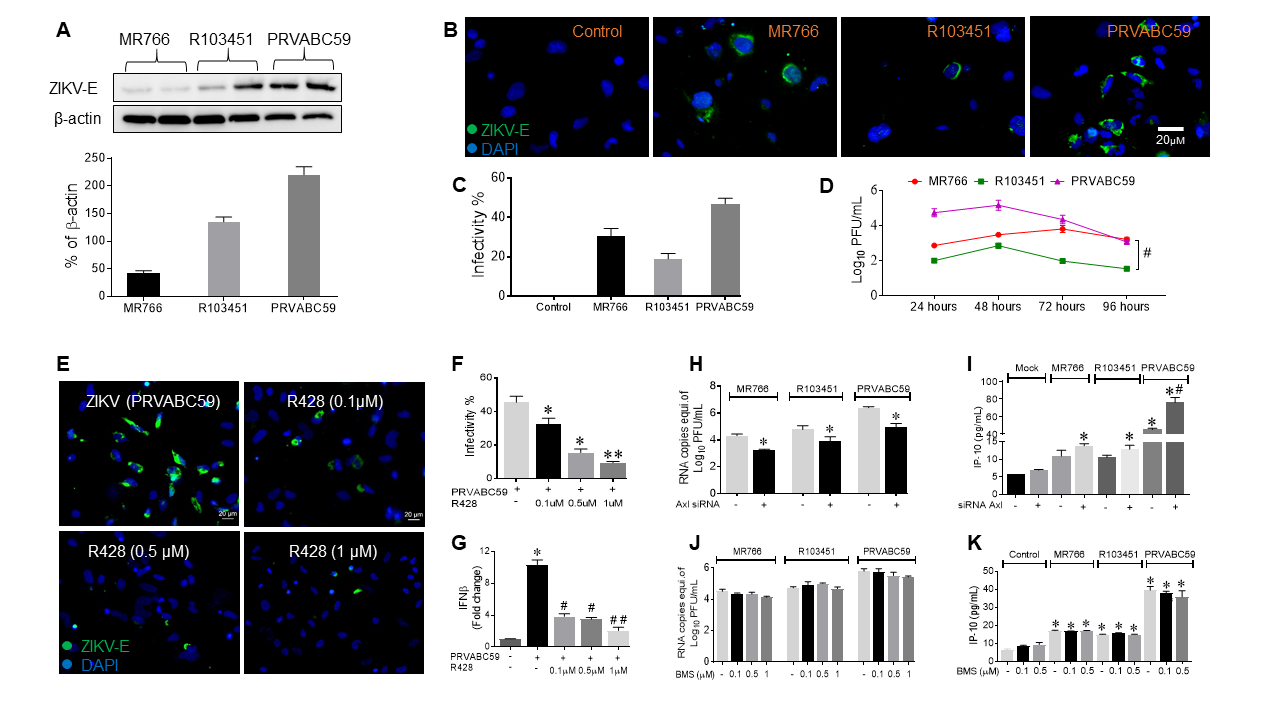

Supplement: S1 Fig — (A) Western blot shows expression of ZIKV-envelope protein in primary human astrocytes infected with three strains of ZIKV (MR766, R103451 and PRVABC59) (B) Representative images of human microglia infected with ZIKV (MR766, R103451 and PRVABC59) for 24 hours and immunolabeled with ZIKV envelope antibody. (C) Percentage infectivity of ZIKV in human microglia measured by immunofluorescence. (D) Viral titers in supernatant of ZIKV infected microglia measured by plaque assay at 24, 48, 72 and 96 hpi. (E and F) TAM inhibitor reduced ZIKV infection in human astrocytes. (E) Representative images of human astrocytes infected with ZIKV (PRVABC59) in presence or absence of R428. (F) Infectivity of human astrocytes measured by immunofluorescence staining. (G) IFN-β response measured by ELISA from cell supernatant of infected astrocytes exposed to increasing concentrations of R428. (H) Viral titers measured by RT-PCR in supernatant of ZIKV infected astrocytes with or without siRNA against AXL. (I) IP-10 secretion measured by ELISA using supernatant of ZIKV infected astrocytes with or without siRNA against AXL. (J) Viral titers measured by RT-PCR using supernatant of ZIKV infected astrocytes with or without exposure of Tyro3 inhibitor (BMS777607). (K) IP-10 secretion measured by ELISA using supernatant of ZIKV infected astrocytes with or without exposure of Tyro3 inhibitor (BMS777607). Mock (PBS) infected cells were used as control and the infection dose of ZIKV was at an MOI of 0.1. Data are presented as mean ± SEM from at least three independent experiments. (* p< 0.05 Vs Control, **p< 0.01 Vs Control # p < 0.05 Vs ZIKV alone). (TIF) [file pone.0208543.s001.tif]

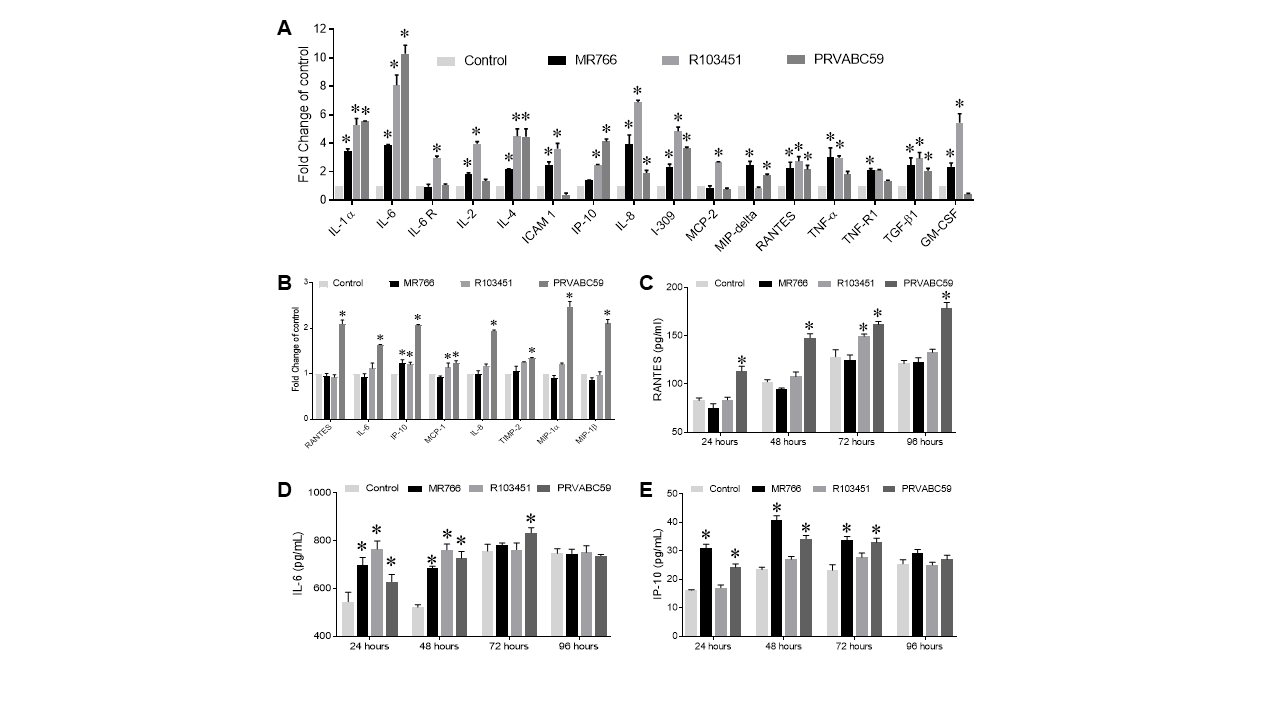

Supplement: S2 Fig — (A) Inflammation was measured using human Cytokine Antibody Array from culture supernatant of ZIKV infected glia. Expression levels are presented as fold increase from control. (B-E) Inflammatory molecules secreted by human microglia infected with three different strains of ZIKV measured by antibody array (B) and ELISA (C-E). Mock (PBS) infected cells were used as control and the infection dose of ZIKV was at an MOI of 0.1. Data are presented as mean ± SEM from at least three independent experiments. (*p< 0.05 Vs Control). (TIF) [file pone.0208543.s002.tif]

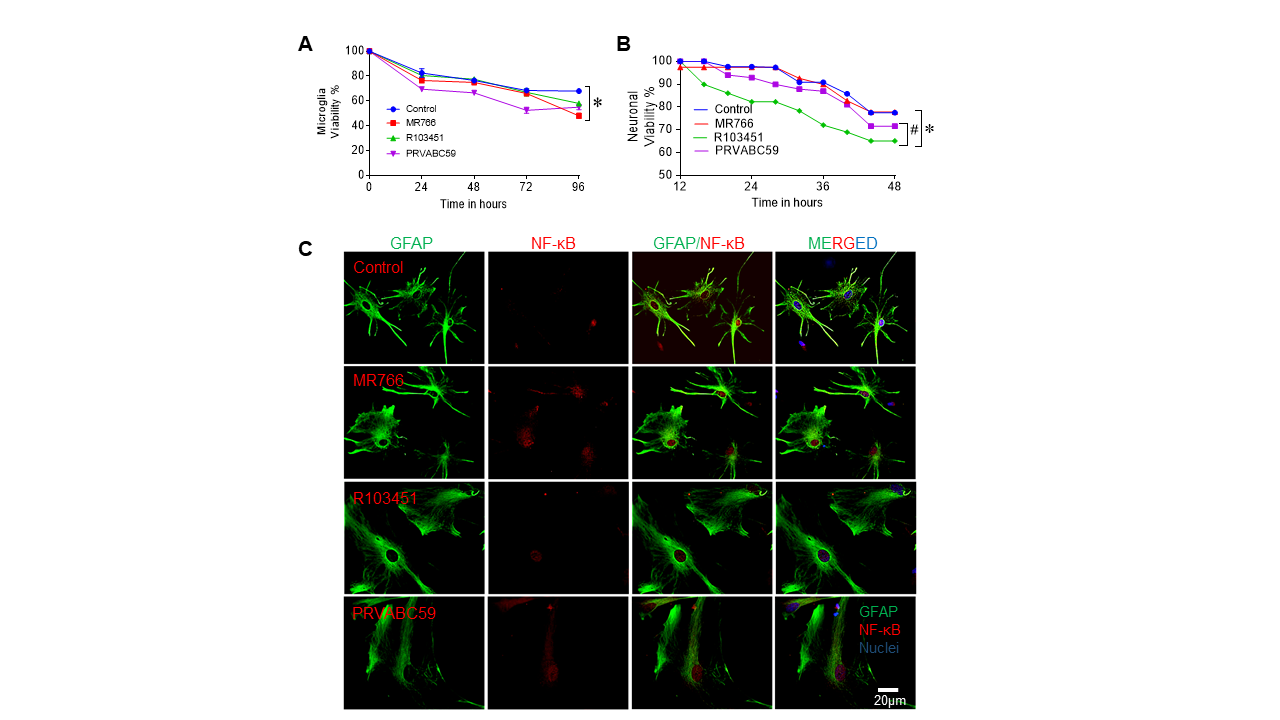

Supplement: S3 Fig — (A) Viability of human microglia at 24, 48, 72 and 96 hpi measured by trypan blue exclusion method. (B) Viability of neurons determined by time lapse image analysis. (C) Immunofluorescence staining of primary human astrocytes with NF-κB, GFAP and DAPI shows both nuclear and cytoplasmic localization of NF-κB. Error bars shown as mean ± SEM from 3–5 separate experiments. Mock (PBS) infected cells were used as control and the infection dose of ZIKV was at an MOI of 0.1. Data are presented as mean ± SEM from at least three independent experiments. (*p< 0.05 Vs Control). (TIF) [file pone.0208543.s003.tif]

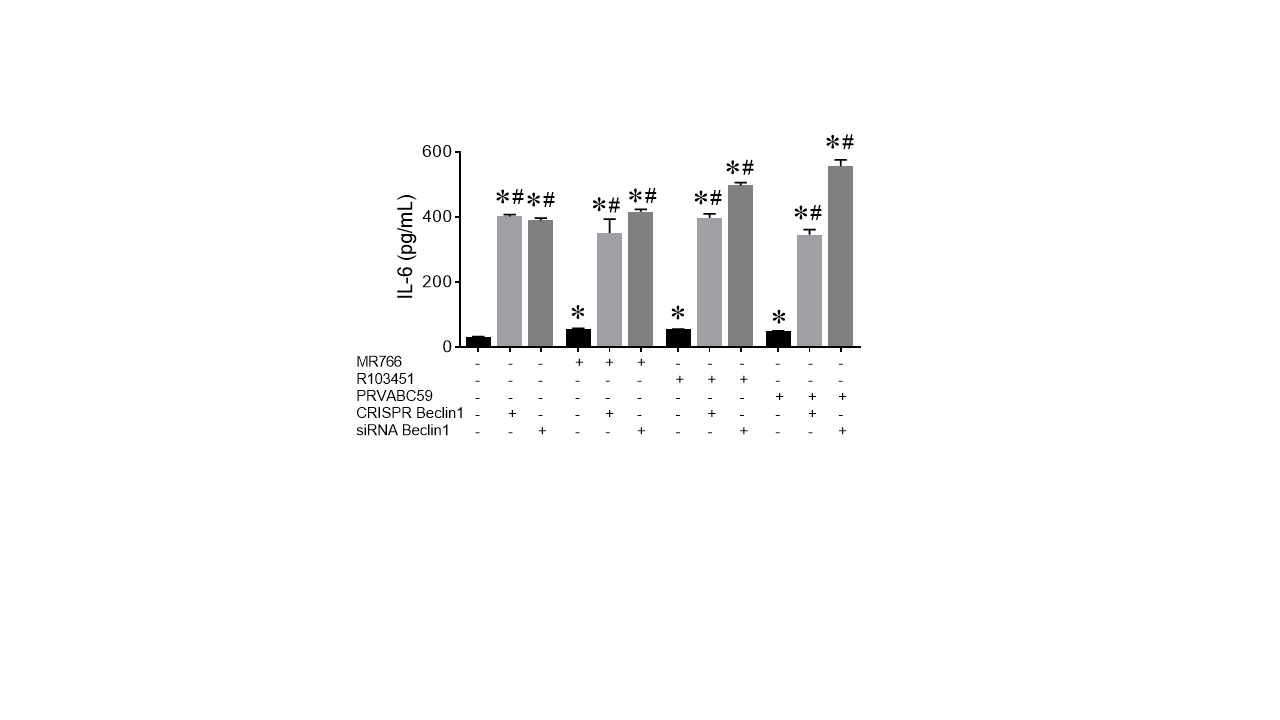

Supplement: S4 Fig — Secretion of IL-6 measured by ELISA using human astrocytes supernatant after 48 hours post infection. Data are presented as mean ± SEM from at least three independent experiments. Mock (PBS) infected cells were used as control and the infection dose of ZIKV was at an MOI of 0.1. Data are presented as mean ± SEM from at least three independent experiments. (*p< 0.05 Vs Control). (TIF) [file pone.0208543.s004.TIF]

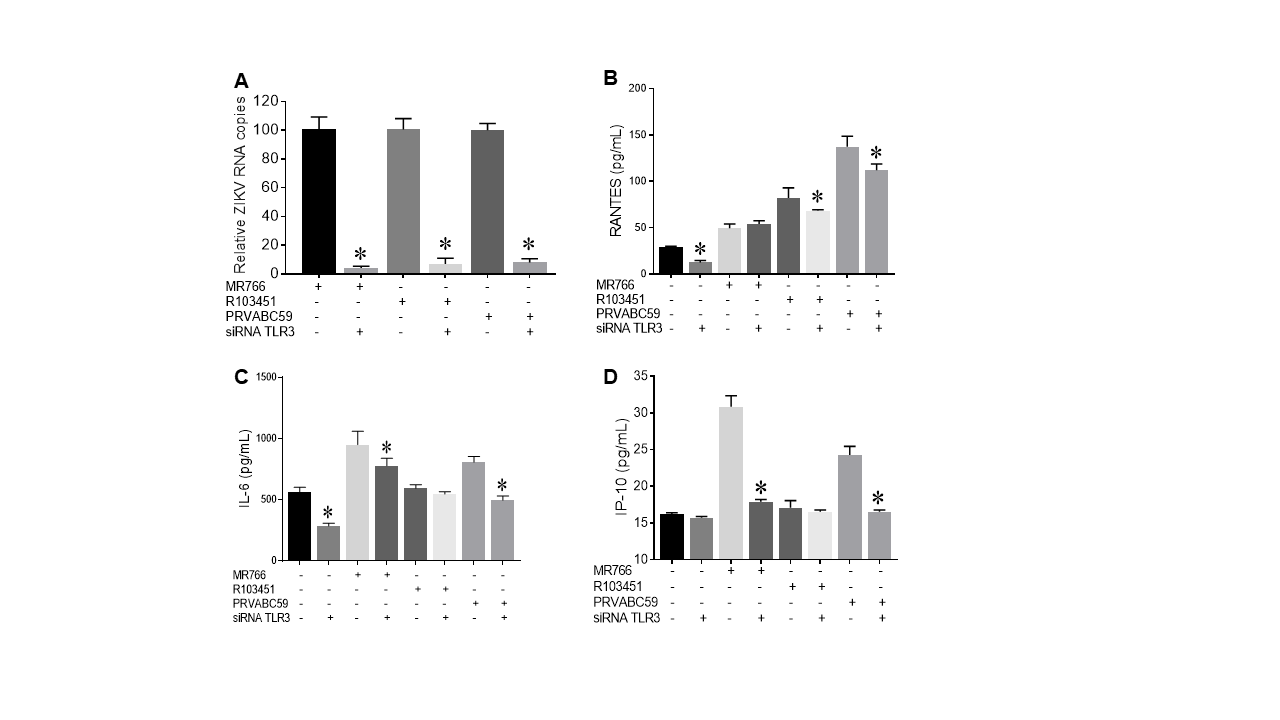

Supplement: S5 Fig — (A) ZIKV titers measured by RT-PCR after 48hpi and TLR3 silencing. (B-D) Inflammatory molecules measured by ELISA after 48 hpi with or without siRNA against TLR3. Mock (PBS) infected cells were used as control and the infection dose of ZIKV was at an MOI of 0.1. Data are presented as mean ± SEM from at least three independent experiments. (*p< 0.05 Vs Control). (TIF) [file pone.0208543.s005.TIF]

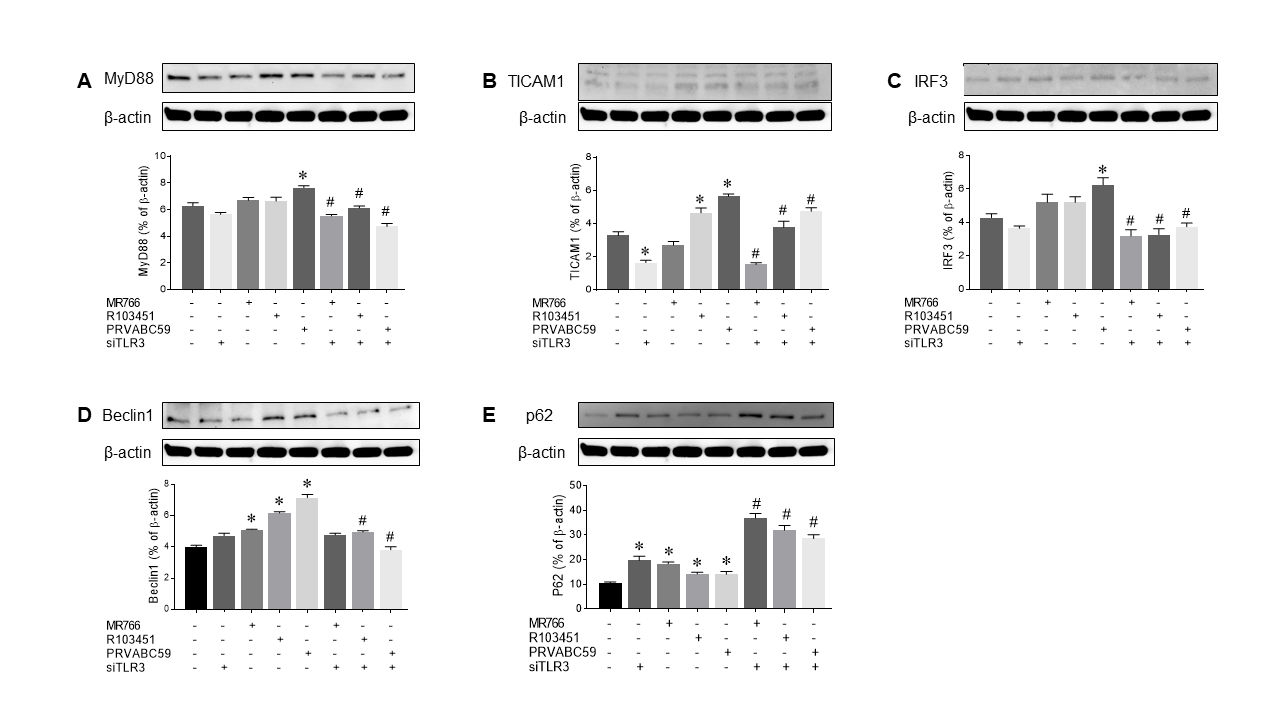

Supplement: S6 Fig — (A-C) Expression of MyD88, TICAM1 and IRF3 (A), Beclin1 (B) and p62/SQSTM1 (C) with and without siRNA against TLR3 as measured by western blot. Mock (PBS) infected cells were used as control and the infection dose of ZIKV was at an MOI of 0.1. Data are presented as mean ± SEM from at least three independent experiments. (*p<0.05 Vs control, # Vs ZIKV alone). (TIF) [file pone.0208543.s006.tif]
