# Supplementary material for: Toll-like receptor 3 regulates Zika virus infection and associated host inflammatory response in primary human astrocytes
Source: PLoS One. 2019 Feb 8;14(2):e0208543. doi: 10.1371/journal.pone.0208543 (PMC6368285; doi:10.1371/journal.pone.0208543)
Supplement: S1 File — (DOCX) [file pone.0208543.s007.docx]

**S1 File. Supplementary materials and methods**

**Microglia and neuronal culture and infection**

Human microglia cell line (HMC3) was purchased from ATCC (Cat. CRL-3304, Manasas, VA, USA) and grown in EMEM supplemented with 10% heat-inactivated FBS. Primary human neurons (Cat. 1520, ScienCell, Carlsbad, CA, USA,) were cultured according to the manufacturer’s instructions in the neurobasal medium (Cat. 1521, ScienCell, Carlsbad, CA, USA,) containing fetal bovine serum and antibiotics. The cells were infected using the same protocol and MOI as with the human astrocytes.

**Microglia and neuronal viability**

Viability of human microglia infected with three different strains of ZIKV was determined by trypan blue dye exclusion method at different time point after infection. For human neuronal viability, time-lapse digital images of neurons were recorded using an inverted microscope with an automated computer-controlled stage encoder and environmental chamber (37 °C, 95% humidity, 5% CO_2_) that allowed repeated tracking of individual neurons after infection over time. Neuronal death was considered to have occurred upon collapse and fragmentation of the cell body.

**NF-κB nuclear localization assay**

Immunofluorescence staining of nuclear factor κB (NF-κB) (Cat. sc-109 (1:200) Santa Cruz Biotech, Santa Cruz, CA, USA) and Glial fibrillary acidic protein (GFAP) (Cat. MAB360 (1: 400), Millipore, Billerica, MA, USA) shows both nuclear and cytoplasmic localization of NF-κB in primary human astrocytes. The numbers of NF-κB puncta within the nucleus and in the cytoplasm were counted manually in 40-60 astrocytes/field using a 40X objective.

**Silencing of TLR3 in microglia**

Human primary microglia were transfected with siRNA against *TLR3* for 24–48 hours using Lipofectamine 2000 (Invitrogen, Carlsbad, CA, USA ) or Fugene HD (Promega, Madison, WI, USA). siRNA and transfection reagent (at a ratio of 1:2) were pre-incubated for 20 min in OptiMEM medium before added to seeded cells. Silencing of TLR3 expression was confirmed by western blot analysis using protein lysates from microglia after 48 hours post-transfection.
